# Switching the Mode of Drug Release from a Reaction-Coupled Low-Molecular-Weight Gelator System by Altering Its Reaction Pathway

**DOI:** 10.1021/acs.biomac.2c01197

**Published:** 2022-12-23

**Authors:** Willem E. M. Noteborn, Sandeepa K. Vittala, Maria Broto Torredemer, Chandan Maity, Frank Versluis, Rienk Eelkema, Roxanne E. Kieltyka

**Affiliations:** † Supramolecular and Biomaterials Chemistry, Leiden Institute of Chemistry, Leiden University, P.O. Box 9502, 2300 RALeiden, The Netherlands; ‡ Department of Chemical Engineering, Delft University of Technology, Van der Maasweg 9, 2629 HZDelft, The Netherlands

## Abstract

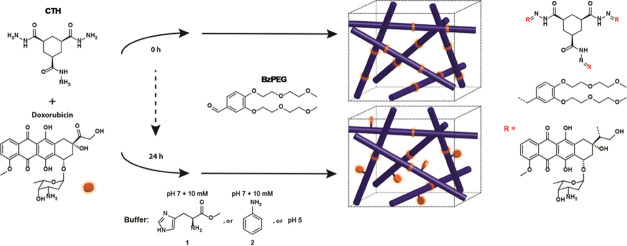

Low-molecular-weight hydrogels are attractive scaffolds for drug delivery applications because of their modular and facile preparation starting from inexpensive molecular components. The molecular design of the hydrogelator results in a commitment to a particular release strategy, where either noncovalent or covalent bonding of the drug molecule dictates its rate and mechanism. Herein, we demonstrate an alternative approach using a reaction-coupled gelator to tune drug release in a facile and user-defined manner by altering the reaction pathway of the low-molecular-weight gelator (LMWG) and drug components through an acylhydrazone-bond-forming reaction. We show that an off-the-shelf drug with a reactive handle, doxorubicin, can be covalently bound to the gelator through its ketone moiety when the addition of the aldehyde component is delayed from 0 to 24 h, or noncovalently bound with its addition at 0 h. We also examine the use of an l-histidine methyl ester catalyst to prepare the drug-loaded hydrogels under physiological conditions. Fitting of the drug release profiles with the Korsmeyer–Peppas model corroborates a switch in the mode of release consistent with the reaction pathway taken: increased covalent ligation drives a transition from a Fickian to a semi-Fickian mode in the second stage of release with a decreased rate. Sustained release of doxorubicin from the reaction-coupled hydrogel is further confirmed in an MTT toxicity assay with MCF-7 breast cancer cells. We demonstrate the modularity and ease of the reaction-coupled approach to prepare drug-loaded self-assembled hydrogels in situ with tunable mechanics and drug release profiles that may find eventual applications in macroscale drug delivery.

## Introduction

Self-assembled biomaterials based on low-molecular-weight gelators (LMWG) possess features such as easy handling and tunability of their properties because of the well-defined nature of the chemical building blocks.^1−5^ Peptides, sugars, nucleosides, or small aromatic molecules self-assemble through noncovalent interactions such as hydrogen bonding, electrostatic, or π–π interactions encoded into the gelator chemical structure to yield gel phase materials.^6−11^ The hydrophobic and hydrophilic regions of the resultant hierarchical assemblies can interact noncovalently with drugs of a matched polarity, dictating the drug release rate through their binding affinity.^12−17^ To further tailor the release profile for drug delivery applications, (reversible) covalent bonding of the drug molecule to the low-molecular-weight gelator can be also pursued, relying on bond hydrolysis under physiological or enzymatic means to trigger drug release.^18−24^ While both approaches share advantages and disadvantages in their preparation and application, the flexibility to alter the drug release profile for a particular gelator is limited, as a commitment to a release strategy is taken early on the molecular design of the gelator.

For most LMWGs, the method of gel preparation influences their supramolecular organization, tremendously impacting the properties of the final material.^25−28^ Environmental conditions such as pH, temperature, or ionic strength of the medium can drive changes in the self-assembly pathway of the LMWG through modulating the balance between kinetics and thermodynamics.^29−34^ A more recent strategy to control the self-assembly pathway involves reaction-coupled assembly, namely, the use of chemical bond formation, change, or disruption *in situ* to trigger the gelation process.^35−41^ More specifically, nongelling precursors outfitted with complementary reactive groups are ligated in the presence of chemical or enzymatic catalysts that provide an additional handle to control the final gel structure and function. Xu and co-workers demonstrated control over the gelation behavior of short peptides through enzymatic catalysis of tyrosine phosphorylation or dephosphorylation reactions by kinases or phosphatases.^42,43^ Alternatively, Eelkema and van Esch showed that tuning the reaction rate of hydrazone bond formation on nongelling precursors using acid or aniline catalysis results in structural changes in the nanofiber networks with consequent effects on gel stiffness.^44,45^

**Scheme 1:**
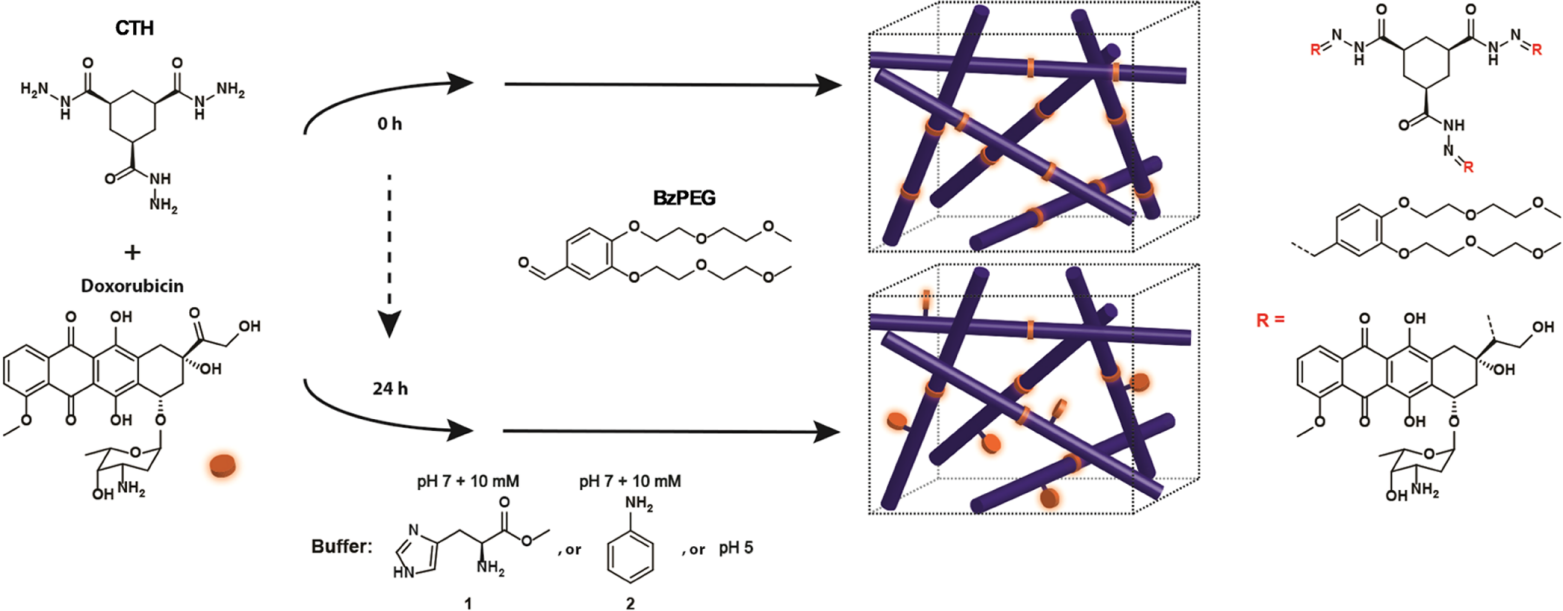
Switching the Mode of Doxorubicin Interaction with a Reaction-Coupled Low-Molecular-Weight Hydrogel by Altering the Reaction Pathway of the Gelator Components, **CTH** and **BzPEG** Preincubation of doxorubicin with CTH for 24 h before the addition of BzPEG leads to a covalently bound drug fraction that affects hydrogel physicochemical properties and its release.

The reaction-coupled approach provides a powerful handle to modulate the structure and physical properties of self-assembled LMWG materials. However, its use in drug delivery is understudied with only a few reports demonstrating noncovalent drug encapsulation.^37,46,47^ Further exploiting the potential to conjugate a drug to the gelator during reaction-coupled self-assembly in situ would provide unique opportunities to tune the release profile at the time of application for various local delivery aims in contrast to earlier approaches that involve exclusive noncovalent or covalent bonding. Thus, we herein examine the effect of altering the reaction pathway of a reaction-coupled gelator on the preparation and application of drug-loaded self-assembled hydrogels where the drug can be covalently bound through a reactive handle. To prepare the drug-loaded hydrogels, we use an acylhydrazone-bond-forming reaction between two nongelling precursors, cyclohexane trishydrazide (**CTH**) and oligo(ethylene glycol)-functionalized benzaldehyde (**BzPEG**), and add doxorubicin that has a ketone moiety that can also react with **CTH** (Scheme 1). Because of the difference in reactivity between the aldehyde of the **BzPEG** and the ketone on doxorubicin, the **CTH** component that contains a hydrazide was first incubated with doxorubicin between 0 and 24 h before the addition of **BzPEG** that triggers hydrogel formation. The addition of **BzPEG** to **CTH** and doxorubicin at 0 h is anticipated to result in noncovalent bonding of the drug, while its addition after 24 h would bias doxorubicin conjugation to the gel network. We thus examine the potential of the reaction-coupled approach to prepare drug-loaded self-assembled hydrogels in situ with tunable mechanical properties and drug release profiles based on the reaction pathway taken.

## Experimental Section

### Materials and Methods

l-Histidine methyl ester (**1**), aniline (**2**), doxorubicin dihydrochloride, dimethylsulfoxide (DMSO), thiazolyl blue tetrazolium bromide (MTT), and Corning Transwell membrane culture inserts (6.5 mm, 0.4 μm pore size) were obtained from Sigma-Aldrich. Dulbecco’s modified Eagle’s cell culture medium (DMEM), fetal calf serum (FCS), penicillin, streptomycin, and GlutaMAX were obtained from Thermo Fisher Scientific. **CTH** and **BzPEG** gelator molecules were synthesized as previously reported.^44^ Water was deionized before use. MCF-7 human breast cancer cells were cultured (37 °C, 5% CO_2_) in DMEM with 10% fetal calf serum, 0.02% GlutaMAX, penicillin (100 U/mL), and streptomycin (100 μg/mL).

LC-MS analysis was performed on a Finnigan Surveyor HPLC system equipped with a Gemini C18 50×4.60 mm column (UV detection at 200–600 nm), coupled to a Finnigan LCQ Advantage Max mass spectrometer with ESI. The mobile phase consisted of H_2_O and CH_3_CN with 0.1% trifluoroacetic acid. UV–vis spectroscopy was executed on a BioDrop μLite using quartz cuvettes of a 1 cm path length. Cell imaging was performed on a LEICA SPE confocal system equipped with a DMI4000B-CS microscope and an HCX APO L U-V-I water immersion objective (40×/NA 0.80). Phase contrast images were acquired on an Olympus IX 81 microscope.

### Hydrogel Preparation

Hydrogels were prepared by separately dissolving the desired concentration of **CTH** (50–100 mM) and **BzPEG** (80–160 mM) in a given buffer as stated below. The same buffer with or without doxorubicin (200 μg) was then preincubated with **CTH** (0–24 h) before adding **BzPEG** so as to result in a final **CTH**–**BzPEG** molar ratio of 1:3 (40:120 mM) unless otherwise stated. Buffers at pH = 7 consisted of 0.1 M phosphate with 0.1 M NaCl and 10 mM of either catalyst **1** or **2**. The buffer at pH = 5 contained 0.1 M phosphate with 0.1 M NaCl.

### Oscillatory Rheology

Oscillatory rheology measurements were obtained on a Discovery Hybrid Rheometer-2 (DHR-2) (TA Instruments) equipped with 20 mm diameter parallel-plate geometry, Peltier-based temperature controller, and a solvent trap. Hydrogel samples were prepared according to the general gelation protocol with and without doxorubicin (0–2.0 mg/mL). The components were mixed by pipetting three times and the sample was loaded on a parallel plate with a 500 μm gap, protected with a solvent trap and a humid environment. Data collection was halted when either a final plateau was reached or when hydrogel samples started to dry out, as observed by changes in the axial force measured by the rheometer. All time sweep measurements were performed at 25 ± 0.2 °C using 0.05% strain and a frequency of 1.0 Hz and executed in triplicate.

### Scanning Electron Microscopy (SEM)

Hydrogel samples (200 μL) were prepared overnight and dehydrated step-wise in ethanol (increasing in ethanol volume; 70–80–90–95–100%, 15 min for each step) and then in acetone. The dehydrated hydrogel was then dried using a Ball-Tec CPD 030 critical point dryer. The dried hydrogel was mounted on a SEM stub and sputter-coated with gold. SEM images were acquired on a JEOL JSM-6400, equipped with a tungsten filament gun operating at 10 kV and 8 mm working distance.

### Determination of the Fractal Dimension

The fractal dimension of the network, *D*_f_, was determined from the rheological data by applying a model based on the Avrami equation
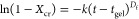
1where *k* is a constant, *X*_cr_ corresponds to the crystallinity of the system, and *D*_f_ is the fractal dimension. The value of *X*_cr_ can be determined from rheological data following the time evolution of the complex modulus, G*, using the equation
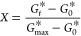
2where *G*_t_^*^, *G*_0_^*^, and *G*_max_^*^ are the complex moduli at time *t*, at the gel point, and at a maximum, respectively. The *D*_f_ was obtained from the slope of the first transition where nucleation and growth of the fibers take place by plotting ln(−ln(1 – *X*_cr_)) against ln(*t* – *t*_gel_). The value of *D*_f_ = 1, 2, 3 indicates the nucleation and elongation of fiber network in a one-dimensional (1D) linear, two-dimensional (2D) platelike, or three-dimensional (3D) spherical fashion.

### Dynamic Light Scattering (DLS)

DLS experiments were performed on a Malvern Zetasizer Nano S using plastic cuvettes with a 10 mm path length and measurements were taken at an angle of 173°. Measurements were performed on 200 μL solutions of aldehyde (120 mM) mixed with and without doxorubicin (1.0 mg/mL) in pH = 7 buffer containing l-histidine methyl ester (10 mM). Their resulting scattered light intensities and corresponding particle sizes were measured at a 173° angle in a polystyrene cuvette at 25 °C. All samples were measured in triplicate.

### Confocal Laser Scanning Microscopy (CLSM)

Confocal laser scanning images were acquired on a Zeiss LSM 710 confocal laser scanning microscope equipped with a Zeiss 40×, 1.3 NA oil immersion objective, using an excitation wavelength of 488 nm and an emission filter of 510–570 nm for imaging doxorubicin-stained hydrogels. Still images were acquired at 536 × 536 pixels (64 μm × 64 μm). Samples were prepared as described in the general gelation protocol. Gel samples were mixed by pipetting before deposition into Ibidi 8-well slides for imaging. Images were taken directly after mixing and 24 h after the onset of gel formation.

### Drug Release Experiments

Hydrogel samples (200 μL) were prepared according to the general gelation protocol with doxorubicin (1.0 mg/mL) using the various buffers at pH = 7 with catalyst **1**. The samples were prepared by the addition of **BzPEG** to the solution containing doxorubicin and **CTH** at 0 h (immediately) and 24 h. A 0.1 M phosphate buffer with 0.1 M NaCl (1 mL) was added on top of the hydrogel and left to stand for 24 h. Afterward, a fraction of the supernatant (800 μL) was replaced daily with the appropriate fresh buffer. The removed fraction was analyzed by UV–vis spectroscopy following the absorbance of doxorubicin at 480 nm to quantify the amount released from the hydrogel. This experiment was also repeated for a hydrogel catalyzed by **1** at pH = 7 with doxorubicin using a release buffer at pH = 5 consisting of 0.1 M phosphate buffer with 0.1 M NaCl.

### Fitting of the Drug Release Data

The cumulative percent drug release over time was fitted with the zero-order, first-order, Higuchi, and Korsmeyer–Peppas models. Zero-order and first-order models correspond to a constant release over time and concentration-dependent release. The equation for the zero-order model is
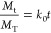
3The equation for the first-order model is
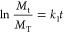
4where *M*_t_ and *M*_T_ are the amount of drug released at time *t* and total drug released, respectively, and *k*_0_ and *k*_1_ are the corresponding zero- and first-order rate constants. The equation for the simplified Higuchi model is
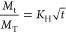
5where *K*_H_ is the kinetic constant and its value is directly proportional to the rate of release. The equation for the Korsmeyer–Peppas model is
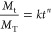
6where *k* is the rate constant and n is the diffusion exponent that determines the mechanism of drug release from the material. Semi-Fickian (*n* < 0.45), Fickian (*n* = 0.45), non-Fickian (0.45 < *n* < 1), and degradation-induced (*n* = 1) release mechanisms can be determined from this model.^65^

### Cell Viability Studies

MCF-7 cells were seeded at a density of 25,000 cells/well (24-well plate) in medium (500 μL) for 24 h before application of the hydrogels. Hydrogels catalyzed by **1** with and without doxorubicin were prepared in Transwell inserts and were suspended the following day in the well plate. The viability of the cells was determined using the MTT assay at 24 h intervals. An MTT stock solution (5 mg/mL) was prepared in 1× PBS and filter-sterilized. The MTT stock solution (20 μL) was added to each well and incubated for 4 h to enable the formation of purple formazan crystals. The medium was aspirated and the formazan crystals were redissolved in DMSO (500 μL). The extent of formazan production was quantified by UV–vis spectroscopy using the absorbance band at 570 nm. Results were reported in % viability = [*A*_570_ (cells treated with hydrogel)/*A*_570_ (cells untreated)] × 100%. All conditions were tested in triplicate. For images taken by confocal laser scanning microscopy, cells were seeded on top of a glass coverslip within the well and removed prior to imaging on a microscope slide.

## Results and Discussion

### Preparation of **CTH**–**BzPEG** Gels under Physiological Conditions

**Figure 1:**
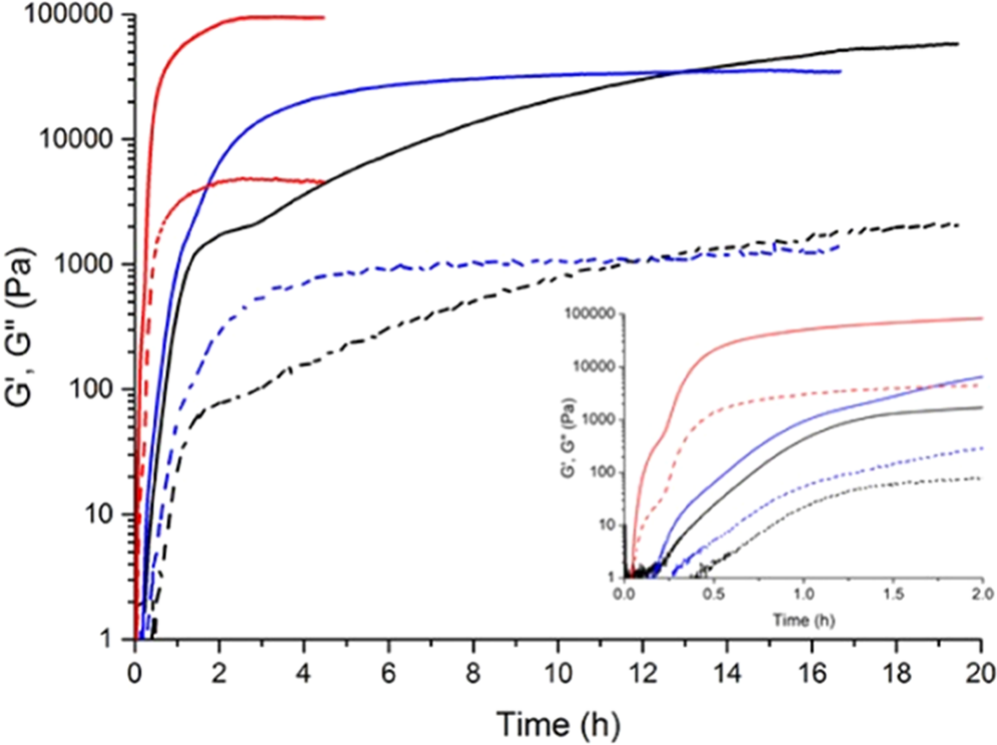
Time sweep measurements (0.05% strain, 1.0 Hz) monitoring hydrogel formation of a 1:3 ratio of **CTH** and **BzPEG** with the various catalysts using oscillatory rheology; **1** (*black*: *G*′ solid, *G*″ dotted), **2** (*blue*: *G*′ solid, *G*″ dotted), pH = 5 (*red*: *G*′ solid, *G*″ dotted). Inset: zoom-in of the first 2 h of data collection showing the two-stage growth profile of the hydrogel samples.

Neutral pH conditions foster the slow reaction between an aldehyde and a hydrazide to form a hydrazone bond on the order of hours; however, organocatalysts can speed up this reaction to minutes.^48−51^ Hence, earlier reports showed that the rate of gelation based on hydrazone bond formation between the **CTH** and **BzPEG** components increased when the pH was reduced to 5 or through the use of nucleophilic aniline, or indoline-based organocatalysts at pH = 7.^52,53^ Because of our eventual interest in applying the reaction-coupled hydrogels as scaffolds for drug delivery, we sought to use l-histidine methyl ester as an organocatalyst as the imidazole moiety is also found in other catalytic amine buffers and lacks toxicity towards cells.^54^ Using oscillatory rheology, we benchmarked the l-histidine methyl ester catalyst against the earlier reported aniline **2** or acid (pH = 5) catalyst to prepare **CTH**–**BzPEG** hydrogels. Dissolution of all gel constituents (**CTH** and **BzPEG**) was afforded in phosphate buffer at pH = 7 or at pH = 5 with catalyst **1** or **2** (10 mM). **CTH** and **BzPEG** were applied in a 1:3 (40:120 mM) molar ratio to give rise to the hydrogels probed in this study unless otherwise indicated. Time sweep measurements enabled tracking of the gelation process by monitoring the increase of the storage (*G*′) and loss (*G*″) moduli as a function of time (Figure 1). The **CTH**–**BzPEG** hydrogels displayed a two-stage growth profile at pH = 7 that can be correlated with the nucleation and growth of nanofibers and their subsequent interconnection (*vide infra*). Comparing *G*′ values at the plateau of the curves with respect to the various catalysts, catalyst **1** (*G*′ = 60 kPa) yielded a stiffer hydrogel than **2** (*G*′ = 35 kPa) at pH = 7. In contrast, an even stiffer material formed at pH = 5 (*G*′ = 95 kPa).

The use of a biocompatible catalyst, l-histidine methyl ester **1**, is compared against earlier used catalysts **2** or pH = 5 on hydrogel formation at pH = 7. Interestingly, the measured *G*′ for the catalysts at pH = 7 showed **1** to be more active than the nucleophilic aniline catalyst **2** in the acylhydrazone reaction, further emphasizing the importance of reaction rate on the gel stiffness. Increasing the amount of **BzPEG** from 3 to 9 molar equivalents (120 mM to 360 mM) in the presence of catalyst **1** reduced the reaction time from 16 to 1.5 h (Figure S1) and resulted in an increase in *G*′.

**Figure 2:**
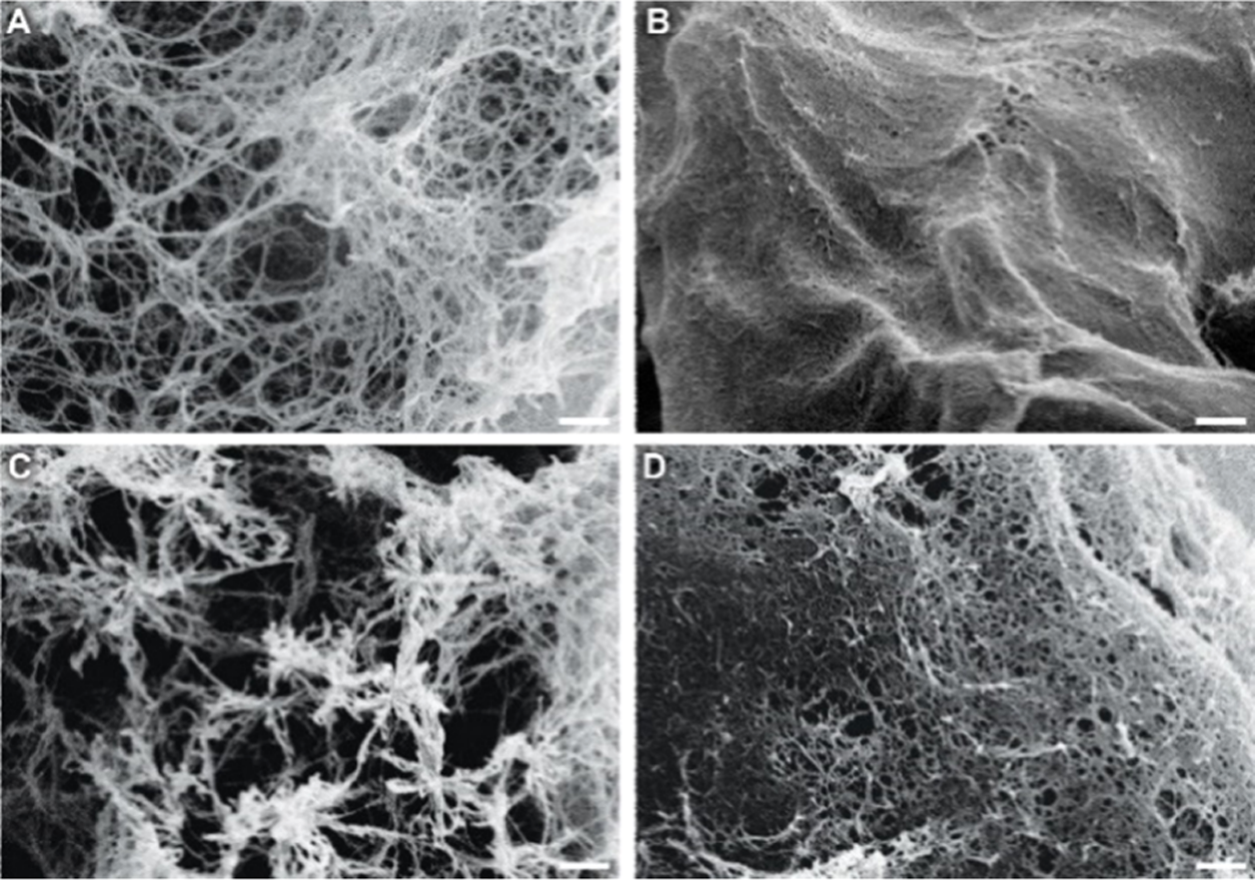
SEM images of **CTH**–**BzPEG** (1:3) hydrogels (A, C, D) catalyzed by (A) **1**, (C) **2**, or (D) pH = 5. (B) SEM image of a **CTH**–**BzPEG** (1:9) hydrogel catalyzed by **1** at pH = 7. Scale bar: 1.0 μm.

We subsequently imaged the self-assembled **CTH**–**BzPEG** gels by scanning electron microscopy (SEM) to better understand the observed differences in the rheology imparted by the various catalysts. Gels consisting of a 1:3 ratio of **CTH** and **BzPEG** with catalyst **1** resulted in an open, interconnected network structure (Figure 2A). Increasing **BzPEG** 3-fold with an equivalent amount of **CTH** led to a densely packed fiber network in line with a stiffer gel (Figure 2B). Compared to **1**, hydrogels prepared using catalyst **2** showed a more open network of fibers, consistent with oscillatory rheology data that presented a lower *G*′ value (Figure 2C). In contrast, gels prepared at pH = 5 displayed a highly dense network of fibers in comparison to the other two catalysts and showed the most rapid gelation profile and highest *G*′ (Figure 2D). These results support a strong relationship between the rate of catalysis and hydrogel stiffness based on the structural presentation of the underlying fiber networks. While a 2-fold excess of **BzPEG** to **CTH** resulted in the stiffest gels, small-molecule aldehydes exert cellular toxicity,^55^ and thus, we employed a 1:3 ratio of the **CTH**–**BzPEG** components for further experiments.

### Altering Doxorubicin Presentation in **CTH**–**BzPEG** Gels

**Figure 3:**
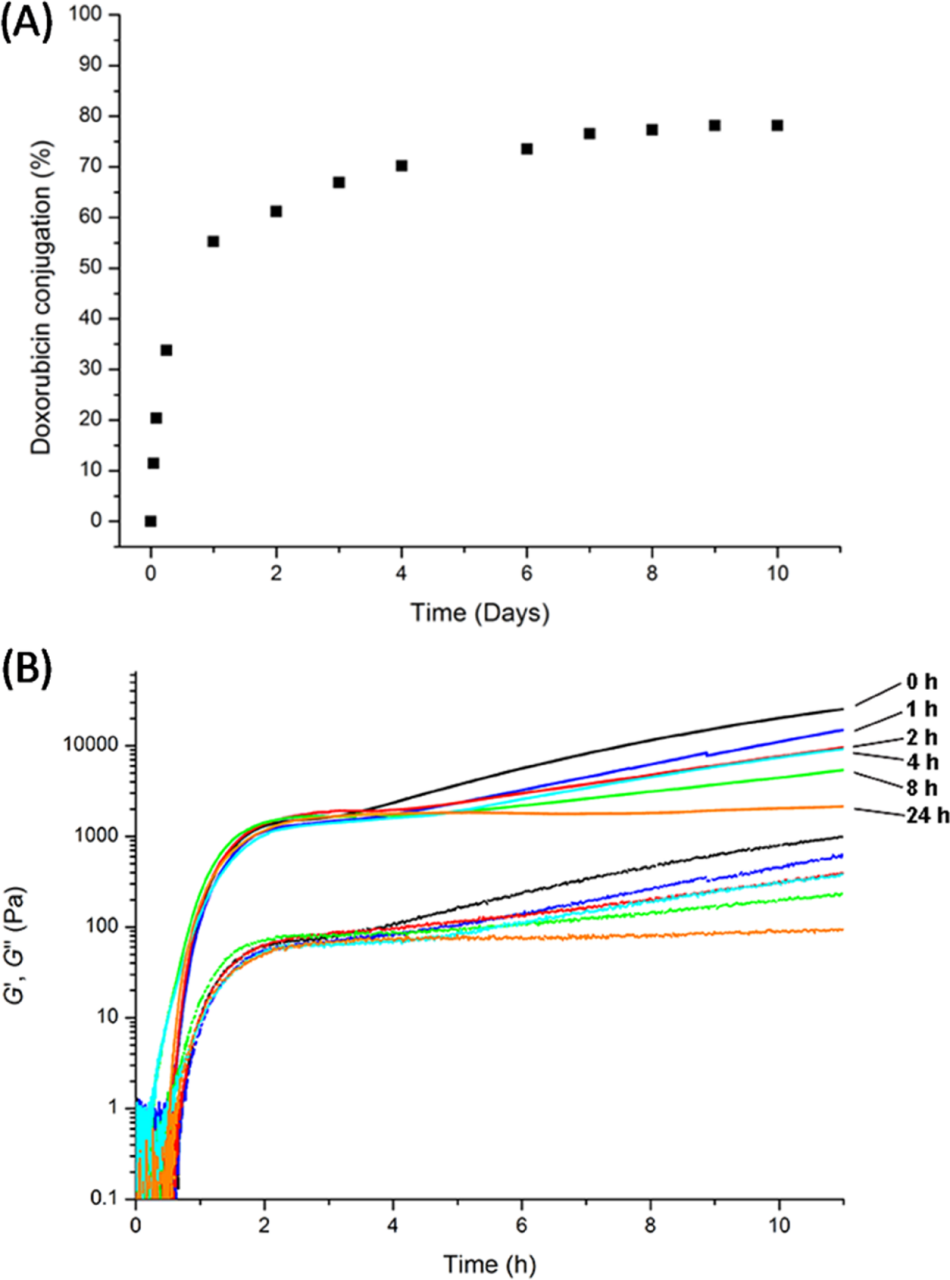
(A) Extent of doxorubicin conjugation (1.0 mg/mL) to **CTH** (40 mM) determined from the ratio of integrated peaks in LCMS spectra corresponding to the unconjugated and conjugated doxorubicin fraction (λ_abs_: 480 nm, *t*_unconjugated_ = 4.55–5 min and *t*_conjugated_ = 3.3–4.45 min). (B) Oscillatory rheology time sweep data of **CTH**–**BzPEG** (1:3) hydrogels loaded with doxorubicin (1.0 mg/mL) and catalyzed by **1**. **BzPEG** was added to a solution containing **CTH** and doxorubicin after 0 h (black), 1 h (blue), 2 h (red), 4 h (cyan), 8 h (green), and 24 h (orange) (*G*′ solid lines, *G*″ dashed lines).

After establishing the preparation of the reaction-coupled hydrogel consisting of **CTH** and **BzPEG** at pH = 7 by catalyst **1**, we further examined the modularity of this chemical strategy to incorporate and release a therapeutic. We selected doxorubicin, a drug that contains an aliphatic ketone and is fluorescent, and probed the addition of **BzPEG** at different times during reaction-coupled gelation, from 0 to 24 h, to alter doxorubicin incorporation and release from the hydrogel network. Because of the slower rate of reaction of the doxorubicin ketone with the hydrazide in **CTH** in contrast to the aldehyde of **BzPEG**, we anticipated that the simultaneous addition of all components would result in noncovalent bonding of doxorubicin to supramolecular fibers of the hydrogel. Increasing the incubation time of doxorubicin with **CTH** prior to the addition of **BzPEG**, on the other hand, would increase the covalently bound drug fraction in the mixture. Thus, we collected LC-MS spectra over time to estimate the amount of doxorubicin covalently bound to the **CTH** core using catalyst **1** at pH = 7. An incubation time of 24 h yielded 55% of the doxorubicin **CTH** product and increased up to 80% after 7 days (Figure 3A).

We further quantified the effect of doxorubicin conjugation on the stiffness of the **CTH**–**BzPEG** network using oscillatory rheology. Time sweep measurements revealed that the addition of **BzPEG** either at 0 or 24 h after **CTH** incubation with doxorubicin (1.0 mg/mL) reduced gel stiffness (Figure 3B). More specifically, we recorded a decrease in *G*′ from 60 kPa for the native **CTH**–**BzPEG** hydrogel to 26 kPa on the addition of **BzPEG** simultaneously with doxorubicin at *t* = 0 h. *G*′ remained constant for this pathway even with varying the concentration of doxorubicin from 0.5 to 2.0 mg/mL (Figure S2). In contrast, preincubation of the **CTH** core with doxorubicin for 24 h followed by the addition of **BzPEG** resulted in a more pronounced decrease in stiffness (2 kPa). Further delaying the addition of **BzPEG** to 7 days to maximize doxorubicin conjugation, as determined from earlier LCMS studies, resulted in the formation of a film that could not be redissolved and was excluded from further studies. Thus, the differences in stiffness recorded for the doxorubicin-loaded hydrogels prepared with different incubation times can be linked to the amount of covalently ligated doxorubicin based on the reaction pathway taken.

The gelation process of the **CTH**–**BzPEG** hydrogel starts with the initial liquid-liquid phase separation of **BzPEG** and water where the **BzPEG** droplets serve as nucleation points for fiber growth. When **CTH** is added, dense regions of fibers associated with the initial **BzPEG** droplets can be seen in the final gel microstructure.^56^ Moreover, the implementation of macromolecular cross-linkers in the gel mixture results in perturbation of the droplets as a function of concentration impacting the final gel properties, namely, stiffness. In gels with spherulitic microstructures, pH, enzyme concentration and processing conditions influence their size and number, as well as the resulting gel mechanics.^57,58^ Hence, we probed the influence of adding doxorubicin on the size of the **BzPEG** droplets using dynamic light scattering (DLS). Doxorubicin addition to the gel reaction mixture reduced the size of the **BzPEG** droplets by 20%. Moreover, their size remained stable and their evolution over time was nearly indistinguishable from droplets composed solely of **BzPEG** (Figure S4). Likely, the decreased size of the **BzPEG** droplets after doxorubicin addition decreases the stiffness of the hydrogels, as previously observed for spherulitic gels with smaller domain sizes.^59^

To understand the effect of doxorubicin conjugation on the formation of the **CTH**–**BzPEG** network, we fitted the oscillatory rheology time sweep profiles with a model based on the Avrami equation (eq 1).^60,61^ The slope of the first transition in the time sweep correlates with the nucleation and fractal growth of the nanofiber network described by analyzing the fractal dimension, *D*_f_.^62^ The **CTH**–**BzPEG** gel in the absence of doxorubicin possesses a *D*_f_ value of 2.07, confirming the fractal-like growth of the fibers into a continuous network. Incubation of doxorubicin with **CTH** and delaying **BzPEG** addition resulted in decreased *D*_f_ values of 1.51 (0 h), 1.46 (1 h), 1.47 (2 h), 1.56 (4 h), 1.27 (8 h), and 1.34 (24 h). The lower *D*_f_ value upon doxorubicin incorporation suggests a decrease in fiber branching and interconnectivity of the network, and is in line with the observed decrease in *G*′ of the hydrogels prepared by delaying **BzPEG** addition from 0 to 24 h. Moreover, longer doxorubicin incubation times resulted in larger differences in the second stage of the rheological profiles between samples. With later addition of **BzPEG** (e.g., 24 h), or a longer doxorubicin incubation step, the second stage of growth in the rheological profile reduced significantly tending to a slope of zero, pointing out that increased conjugation of the drug halts further interconnection of the network that gives rise to an increase in stiffness.

We performed microscopic imaging of the doxorubicin-loaded hydrogels in their dried and hydrated states to better understand the microstructural features behind the observed differences in rheological properties. Scanning electron microscopy (SEM) imaging of the freeze-dried doxorubicin-loaded **CTH**–**BzPEG** hydrogels exhibited a densely packed network of nanoscale fibers for samples where **BzPEG** was added at 0 or 24 h (Figure S5). Both of these gels exhibited similar features to SEM images of the **CTH**–**BzPEG** gel formed with **1** at pH = 7 lacking doxorubicin. The fluorescence of doxorubicin also permitted imaging of the self-assembly process of the **CTH**–**BzPEG** gelator in water by confocal laser scanning microscopy (CLSM) using an excitation wavelength of 480 nm (Figure S6). Time-lapse imaging of the self-assembly process of the **CTH**–**BzPEG** hydrogel every 10s displayed the formation of fluorescent **BzPEG** droplets with an average size of 1.38 ± 0.78 μm (Figure S6). This result confirms their interaction with doxorubicin, and further substantiates the decrease in droplet size found in DLS measurements. Subsequently, depletion of the **BzPEG** droplets occurred on reaction-coupled self-assembly and fluorescent fibers from the droplets emerged that eventually produced a dense interconnected network (Figure S7 and Movie S1). The doxorubicin colocalized with the **CTH**–**BzPEG** fibers in the self-assembled state, confirming its interaction either in a covalent or noncovalent mode. These results agree with earlier measured oscillatory rheology data that showed a two-stage growth process.

### Doxorubicin Release Varies with Mode of LMWG Assembly

We further evaluated the consequence of altering the reaction pathway of the **CTH**–**BzPEG** hydrogel on the rate and mechanism of doxorubicin release. In a typical experiment, a release buffer (5× the gel volume) composed of PBS at pH = 7 (or pH = 5) was layered on top of the doxorubicin-loaded gels and exchanged every 24 h to quantify the amount of released drug by measuring absorbance at 480 nm. For the pH = 7 condition, we compared doxorubicin release from the hydrogels prepared by the two different reaction pathways where **BzPEG** was added at 0 and 24 h. Application of a pH = 5 buffer for a period of 7 days to a gel with **BzPEG** addition at 0 h resulted in an increase of the release rate compared to pH = 7 due to the more favorable hydrolysis of the acylhydrazone bond at acidic pH (Figure S8).

We then fitted the cumulative drug release over time with several models (e.g., zero order, first order, Higuchi and Korsmeyer–Peppas) to better understand the consequence of the selected reaction pathway on the doxorubicin release mechanism.^46,63^ The nonlinearity of both drug release profiles (**BzPEG** addition at 0 and 24 h) led to a poor fit of the zero and first-order models (Figure S9). Thus, we applied the Higuchi model that describes drug release from a solid matrix to the doxorubicin release data (Figure S10). The dissolution constant *K*_H_ represents the rate of drug release, which can be modified in response to stimuli (e.g., temperature, pH) and physical features of the carrier that alter drug diffusion.^63,64^ In the first step of the release profile (step 1), both conditions (0 and 24 h) exhibited a slight difference in slope or *K*_H_. The release rate of the drug from the hydrogel where **BzPEG** was added at 24 h was slightly greater (15%) than at 0 h and can be attributed to differences in the self-assembled hydrogel networks as evidenced by their distinct stiffnesses (26 kPa (0 h) to 2 kPa (24 h), Figure 3B). Conversely, in the second step (step 2) of the release profile the hydrogel with **BzPEG** addition at 24 h, where increased covalent conjugation of the drug occurs, displayed a 40% reduction in slope, and thus *K*_H_, compared to its addition at 0 h (Table 1). This reduction in *K*_H_ is a consequence of the increased covalent ligation of doxorubicin to the network compared to the unconjugated condition.

**Table 1:** Fitted Parameters from the Higuchi and Korsmeyer–Peppas Models Applied to the Drug Release Profiles from Doxorubicin-Loaded **CTH**–**BzPEG** Hydrogels

	Higuchi		Korsmeyer–Peppas	
**BzPEG** addition time	*K*_H_ (% h^–1^)	*R*^2^	*n*	*R*^2^
0 h (step 1)	2.16	0.99	0.71	0.99
0 h (step 2)	1.56	0.99	0.47	0.99
24 h (step 1)	2.52	0.98	0.63	0.98
24 h (step 2)	0.92	0.99	0.29	0.99

**Figure 4:**
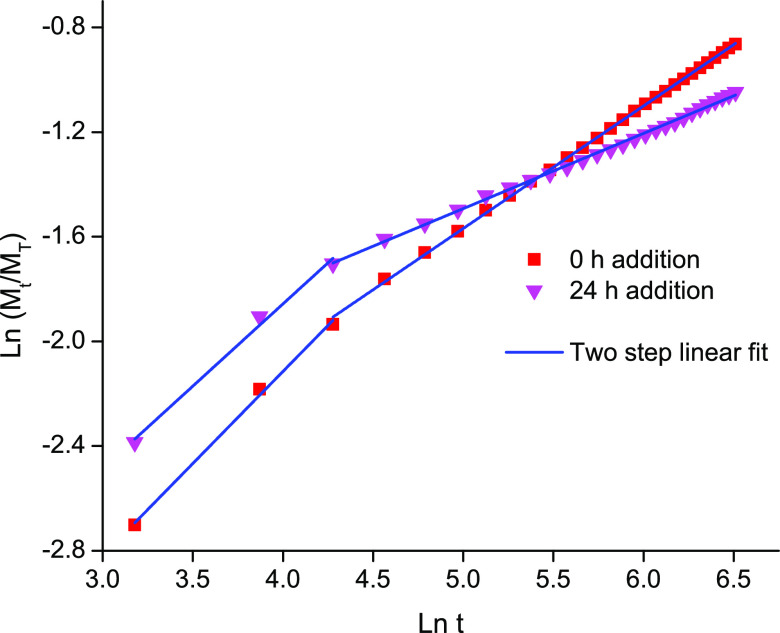
Fitting of the Korsmeyer–Peppas model to the doxorubicin release profile from **CTH**–**BzPEG** hydrogels where **BzPEG** is added at *t* = 0 h (red square) or 24 h (pink triangle).

To pinpoint the release mechanism for the individual steps, we applied the Korsmeyer–Peppas model that describes drug release from a polymeric matrix. Both **BzPEG** conditions (0 and 24 h) (Figure 4 and Table 1) exhibited a diffusion exponent *n* > 0.45 in step 1, indicating non-Fickian diffusion or anomalous transport of the drug through the matrix.^65^ This mechanism of release likely arises from the simultaneous initial swelling of the hydrogel in response to the supernatant buffer added and Fickian diffusion of noncovalently bound drug. In the second stage (step 2) of the release profiles, we obtained *n* values of 0.47 and 0.29 for **BzPEG** addition at 0 and 24 h, respectively, with good regression values (*R*^2^ = 0.98). The *n* value close to 0.45 suggests a Fickian mode of diffusion for the sample with **BzPEG** addition at 0 h, whereas its addition at 24 h yielded an *n* < 0.45 consistent with a semi-Fickian mode involving a superposition of release processes. This switch in the mode of drug release in the second stage follows the reaction pathway of the gelator components. Noncovalent bonding of the drug due to the addition of **BzPEG** at 0 h promotes a Fickian mode of diffusion, whereas its addition at 24 h results in a semi-Fickian mode due to an increased amount of covalently bound drug in the self-assembled gel. The capacity to tune the release mechanism, and thus the rate by altering the reaction pathway of the gel components opens the door to tuning of the drug release profile as needed for eventual local delivery applications.

### Doxorubicin Release from **CTH**–**BzPEG** Gels Impacts Cell Viability

**Figure 5:**
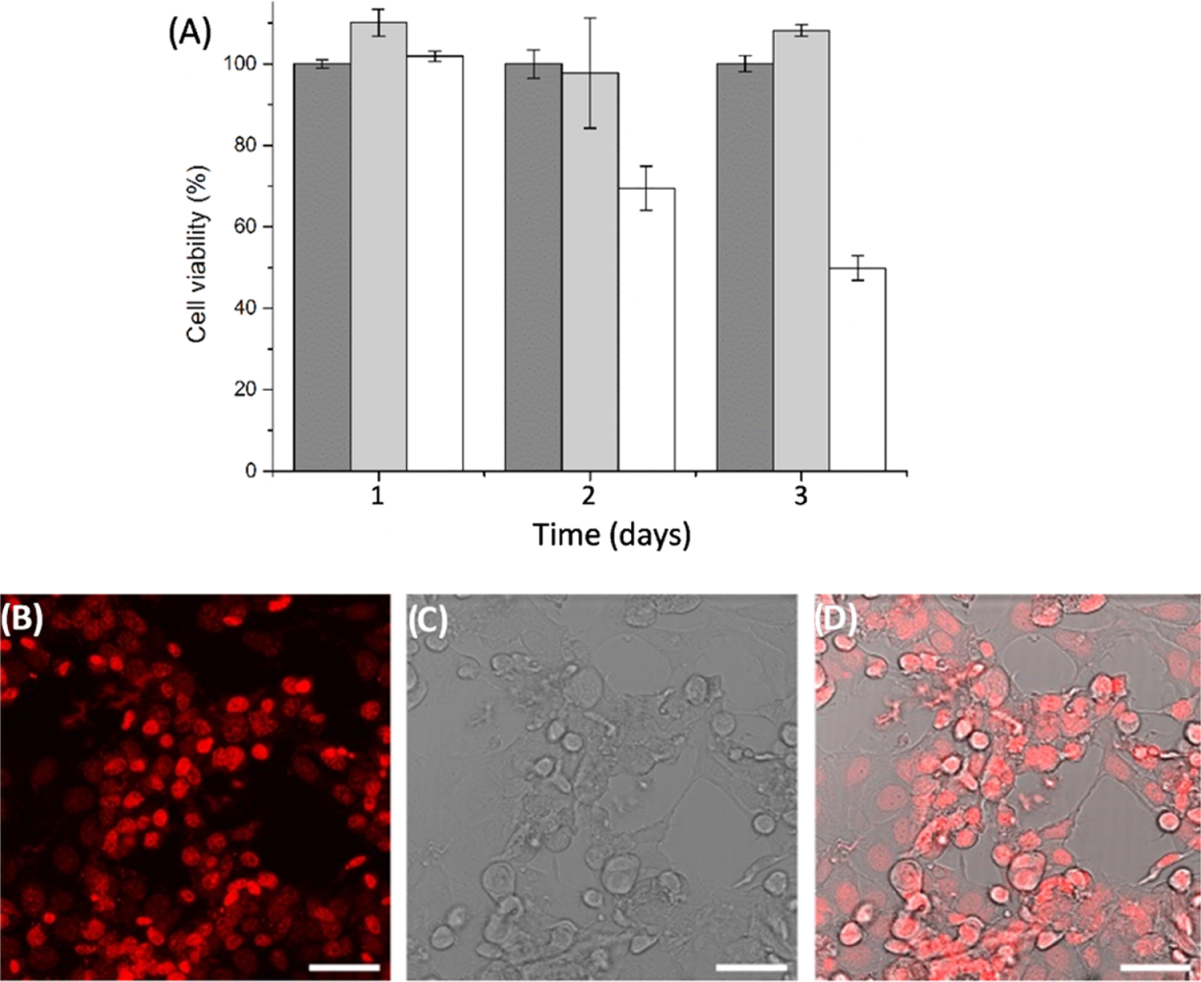
(A) MTT cell viability assay results of MCF-7 cells treated with doxorubicin-loaded (0.5 mg/mL) **CTH**–**BzPEG** hydrogels catalyzed by **1**. Conditions: untreated cells (dark gray); cells treated with **CTH**–**BzPEG** hydrogel (light gray); cells treated with doxorubicin **CTH**–**BzPEG** hydrogels (white). Error bars represent ± s.d. CLSM micrographs of MCF-7 cells subjected to hydrogels with doxorubicin (0.5 mg/mL). (B) Fluorescent image (doxorubicin, λ_ex_ = 480 nm), (C) transmission image, and (D) merged image. Scale bar: 50 μm.

We evaluated the cytocompatibility of the **CTH**–**BzPEG** hydrogel prepared with the l-histidine methyl ester catalyst and the efficacy of doxorubicin delivery by performing an *in vitro* MTT cytotoxicity assay with an MCF-7 human breast cancer cell line. We prepared Transwell inserts loaded with the various hydrogels and positioned them above a cell monolayer in a culture plate, assessing viability up to 3 days post-exposure (Figures 5A and S11). Compared to the control, near-quantitative cell viability in the **CTH**–**BzPEG** hydrogel indicated the suitability of catalyst **1** for cell culture applications. Cells treated with the **CTH**–**BzPEG** hydrogels loaded with doxorubicin and **BzPEG** addition after 24 h showed a reduction in cell viability to 70 and 50% after 2 and 3 days, respectively. The gradual reduction in cell viability supports the slow release of doxorubicin from the **CTH**–**BzPEG** hydrogel matrix, corroborating earlier drug release studies.

We then performed CLSM imaging of MCF-7 cells subjected to the **CTH**–**BzPEG** hydrogels loaded with doxorubicin after **BzPEG** addition at 24 h to visualize doxorubicin uptake and induced cell death (Figure 5B). Internalization of doxorubicin resulted in bright red emission within the cell and simultaneous morphological changes consistent with cell death. These results demonstrate the suitability of catalyst **1** for cell culture applications and the effective slow release of doxorubicin from the reaction-coupled hydrogel.

## Conclusions

We demonstrate control over the release of a drug, doxorubicin, from a reaction-coupled LMWG by modifying its reaction pathway and thus, its mode of bonding and self-assembly to form gel phase materials in water. l-Histidine methyl ester, **1**, showed effective catalysis of the acylhydrazone-bond formation to prepare the gelator at pH = 7, resulting in stiffer gels compared to aniline **2**. We then exploited the modular nature of the reaction-coupled approach to prepare doxorubicin-loaded self-assembled hydrogels altering the reaction pathway of **CTH** and **BzPEG**, adding doxorubicin first to **CTH** and then **BzPEG** either at 0 or 24 h. Incorporation of doxorubicin into the **CTH**–**BzPEG** network decreased the stiffness of the gels, as a consequence of its interaction with the **BzPEG** droplets at the start of reaction-coupled self-assembly, and further reduced fiber branching processes necessary to facilitate network interconnectivity at the microscale. With increased covalent ligation due to altering the reaction pathway, the gel network releases doxorubicin in a sustained manner with a decreased slope in the second stage of the release profiles; fitting with the Korsmeyer–Peppas model reveals a switch from a Fickian to a semi-Fickian mode of drug diffusion. Cell viability assays confirm the cytocompatibility of catalyst **1** and support the slow release of doxorubicin from the gel network. This study showcases the modularity and ease of the reaction-coupled self-assembly to prepare drug-loaded hydrogels in situ with mechanics and release profiles that can be tuned by altering the reaction pathway gelator and drug components. The user-defined approach taken here to modulate the properties of the drug-loaded self-assembled hydrogels can be attractive for a range of local drug delivery targets.

## Supporting Information Available

Oscillatory rheology measurements; Avrami analysis; DLS, SEM, and CLSM images; cumulative drug release; fits of cumulative drug release to zero-order, first-order, and Higuchi models; and phase contrast images of MCF-7 cells (PDF)
Time-lapse CLSM images of the **CTH**–**BzPEG** self-assembly (AVI)

## Supplementary Material

bm2c01197_si_001.pdf

bm2c01197_si_002.avi
