# Supplementary material for: Switching the Mode of Drug Release from a Reaction-Coupled Low-Molecular-Weight Gelator System by Altering Its Reaction Pathway
Source: Biomacromolecules. 2022 Dec 23;24(1):377–86. doi: 10.1021/acs.biomac.2c01197 (PMC9832487; doi:10.1021/acs.biomac.2c01197)
Supplement: Supplementary file 1 — bm2c01197_si_001.pdf [file bm2c01197_si_001.pdf]

Supporting information

Switching the mode of drug release from a reaction-coupled low molecular weight gelator system by altering its reaction pathway

Willem E.M. Noteborn,[a‡] Sandeepa K. Vittala,[a‡] Maria Broto Torredemer,[a] Chandan Maity,[b] Frank Versluis,[b] Rienk Eelkema[b] and Roxanne E. Kieltyka*[a]

[a]Supramolecular and Biomaterials Chemistry, Leiden Institute of Chemistry, Leiden University, P.O. Box 9502, 2300 RA Leiden, The Netherlands
[b]Delft University of Technology, Department of Chemical Engineering, Van der Maasweg 9, 2629 HZ Delft, The Netherlands

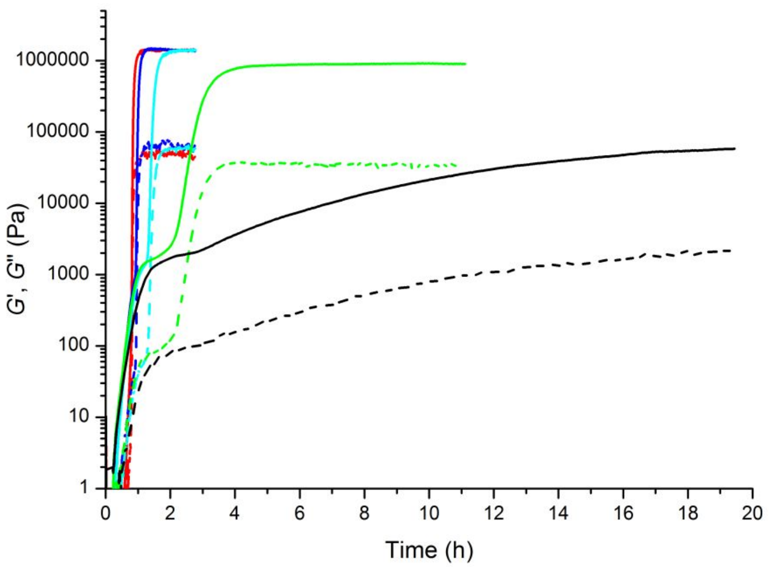

Figure S1: Oscillatory rheology time sweep measurements of 40 mM hydrogels of **CTH** catalyzed by **1** with decreasing equivalents of **BzPEG** (*G*' *solid lines*, *G*" *dashed lines*). **CTH**: **BzPEG** = 1:9 (*red*), 1:6 (*blue*), 1:4.5 (*cyan*), 1:3.75 (*green*), 1:3 (*black*).

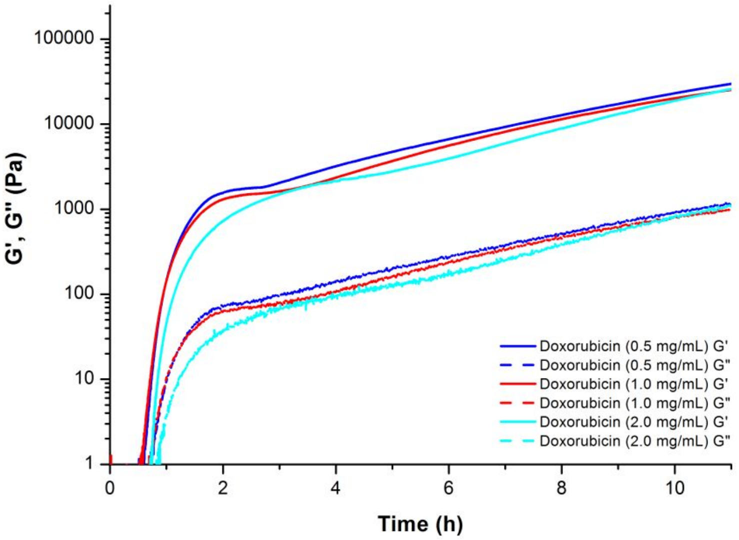

Figure S2: Oscillatory rheology time sweep measurements of **CTH**: **BzPEG** (1:3) hydrogels catalyzed by **1** loaded with varying amounts of doxorubicin (0.5 – 2 mg/mL) without pre-incubation.

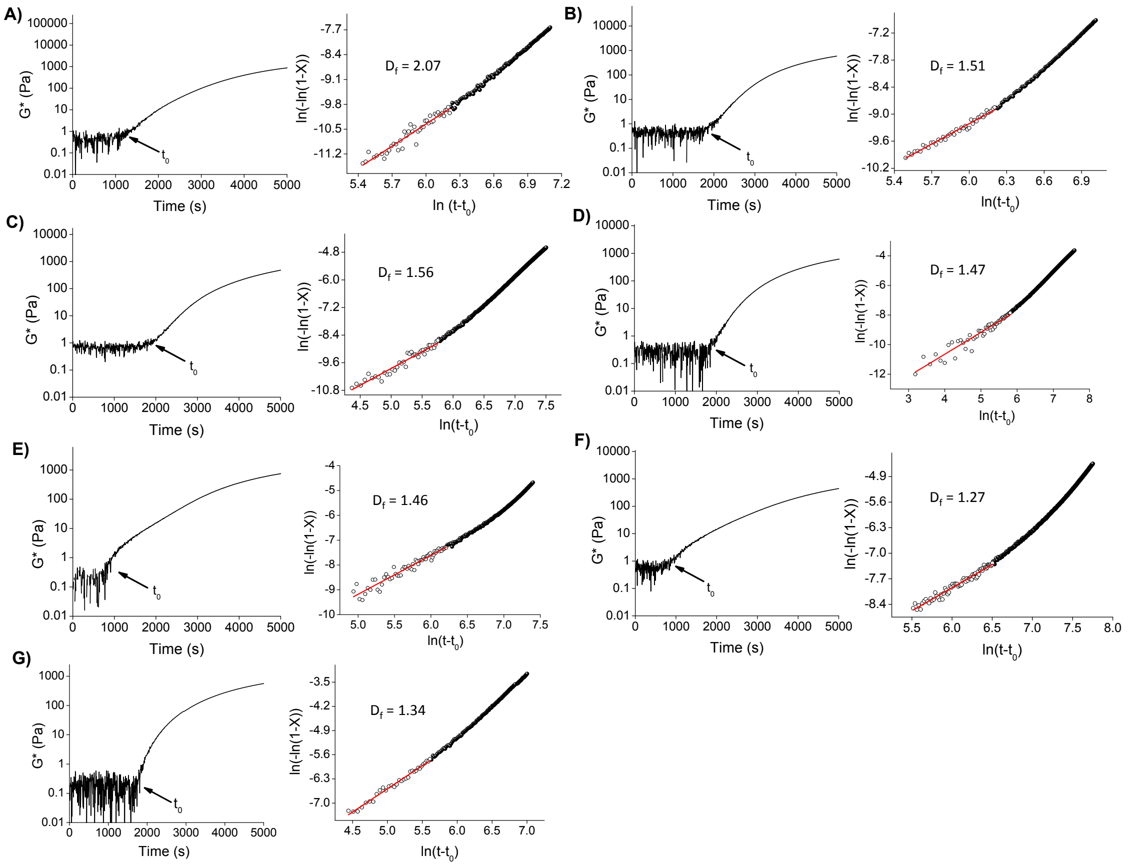

Figure S3: Avrami analysis of complex moduli (G^*) obtained from oscillatory rheology profiles of the various **CTH-BzPEG** hydrogel samples with and without doxorubicin (1 mg/mL) at different incubation times. Time sweep data and Avrami plots of A) gel without doxorubicin, B) 0 h, C) 1 h, D) 2 h, E) 4 h, F) 8 h, and G) 24 h doxorubicin incubation. Arrows indicate the t_0 region of the curve that is used for the subsequent analysis.

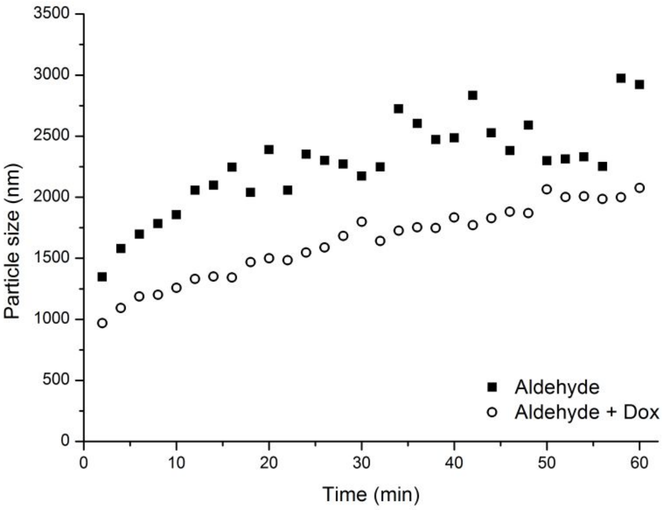

Figure S4: **BzPEG** microparticle stability with (*open circles*) and without (*closed squares*) doxorubicin (1 mg/mL) measured over time by DLS.

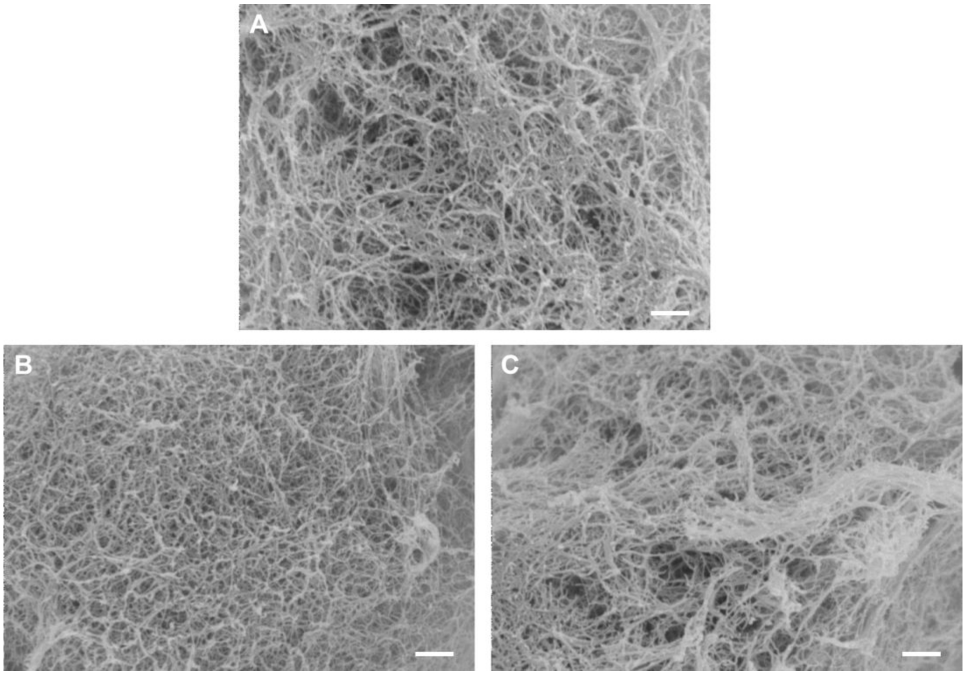

Figure S5: SEM images of **CTH-BzPEG** hydrogels (1:3) catalyzed by **1** at pH = 7, without (A) and with doxorubicin (1 mg/mL) upon addition of **BzPEG** at 0 h (B) and 24 h (C). Scale bars : 1 μm.

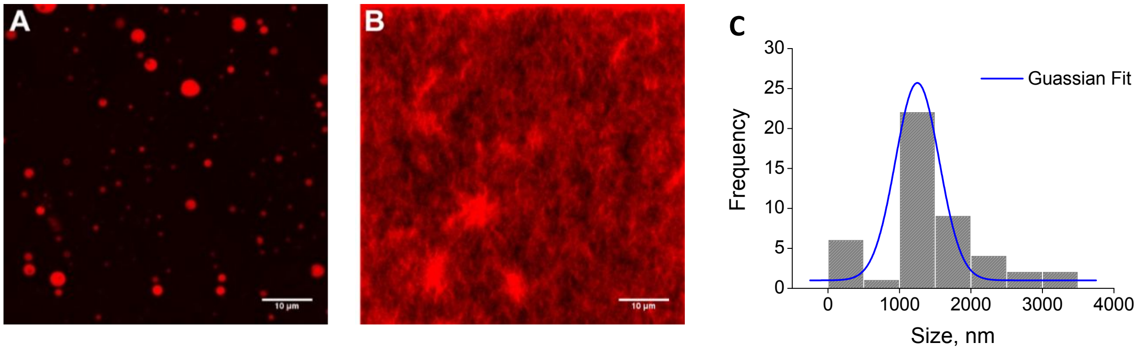

Figure S6: CLSM micrographs of doxorubicin-loaded hydrogels (**CTH-BzPEG** molar ratio = 1:3 catalyzed by **1** at pH 7, doxorubicin (1 mg/mL), **BzPEG** addition at 24 h. The fluorescent properties of doxorubicin (λ_{ex} = 480 nm ; λ_{em} = 510-570 nm) are used for visualization. (A) Doxorubicin interacts with **BzPEG** droplets at the beginning of the gelation process and (B) further colocalizes with the **CTH-BzPEG** hydrogel network after reaction-coupled self-assembly; (C) Statistical distribution of doxorubicin-loaded **BzPEG** particles and its Gaussian fit.

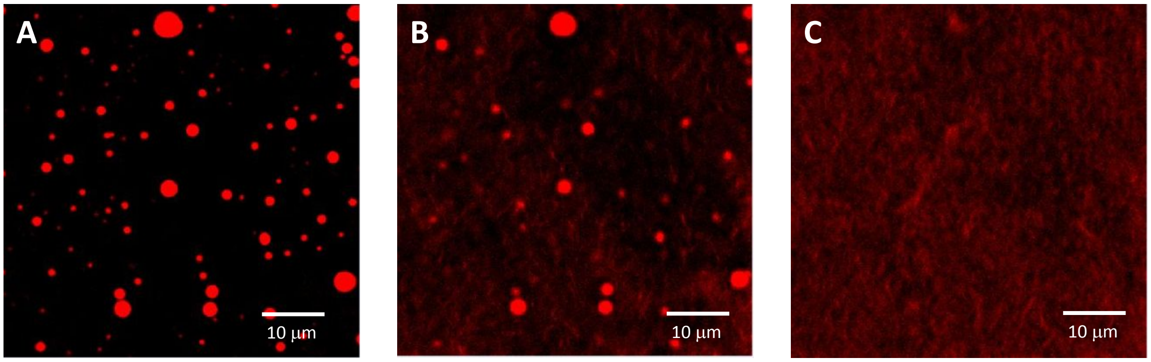

Figure S7: Time-lapsed CLSM images of the **CTH-BzPEG** self-assembly. A) **BzPEG** after incubation of doxorubicin (1 mg/mL) for 24 h; B) 5 min and C) 30 min after addition of **CTH**. λ_{ex} = 480 nm ; λ_{em} = 510-570 nm. Full videos can be found in Movies S1.

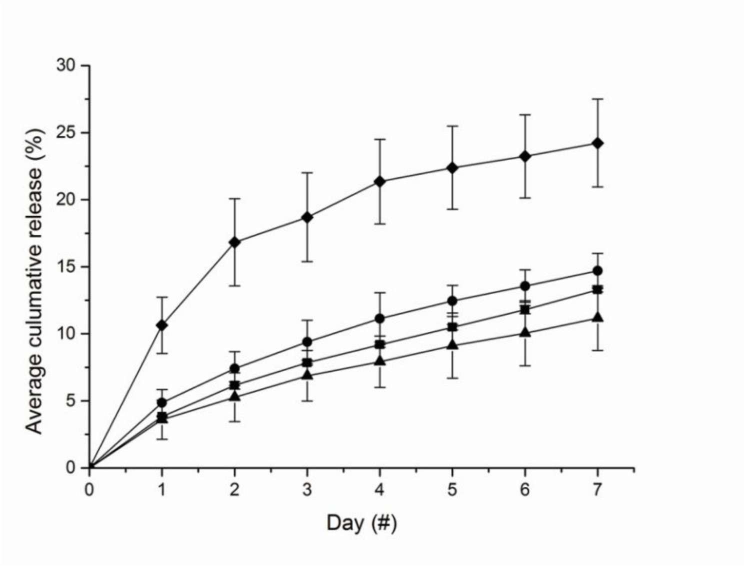

Figure S8: Cumulative drug release (%) of doxorubicin (1 mg/mL) from the **CTH-BzPEG** hydrogel prepared by addition of **BzPEG** at 0 h using catalyst **1** (*square*), **2** (*circle*), pH = 5 (*triangle*) with pH = 7 release buffer. A release buffer of pH = 5 (*diamond*) was also examined for a sample prepared from catalyst **1**.

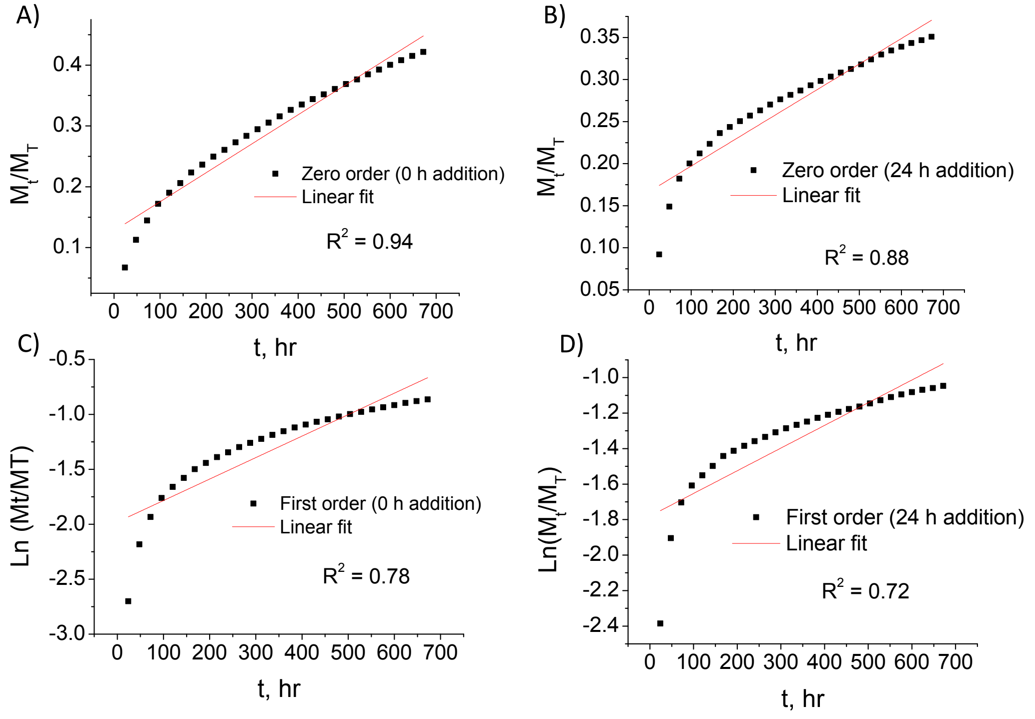

Figure S9: (A-B) Zero order and (C-D) first order fits of cumulative drug release profiles of doxorubicin (1 mg/mL) from **CTH-BzPEG** hydrogels prepared by **BzPEG** addition at 0 h and 24 h.

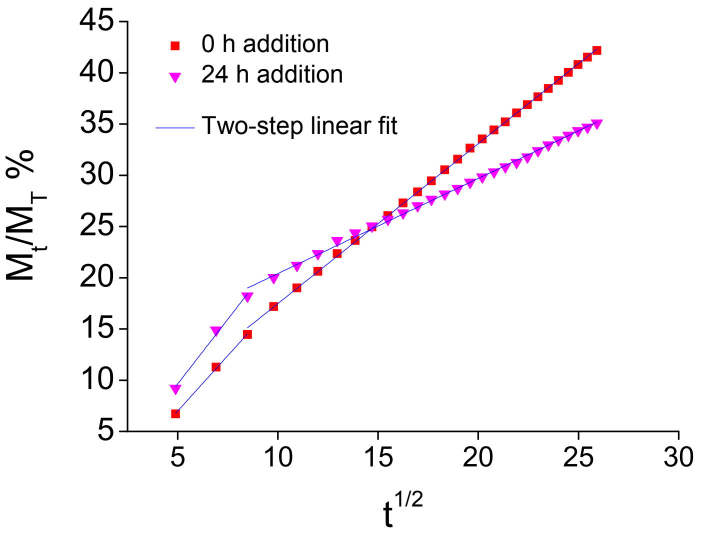

Figure S10: Fitted cumulative drug release profiles of doxorubicin (1 mg/mL) from **CTH-BzPEG** hydrogels prepared by **BzPEG** addition at 0 h and 24 h using a two-step Higuchi model.

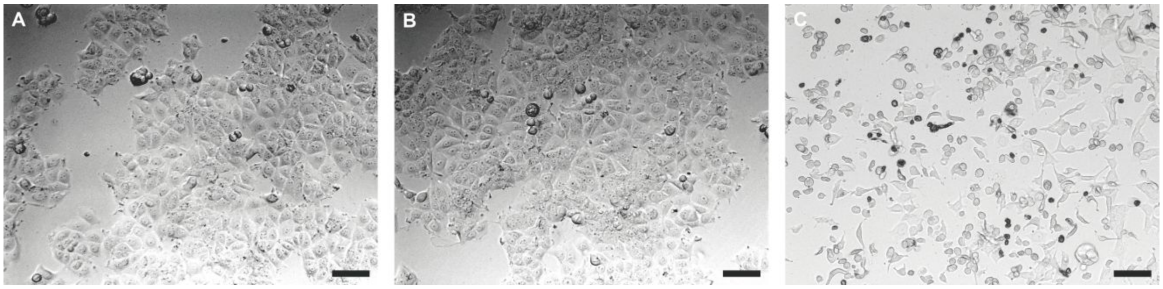

Figure S11: Phase contrast images of MCF-7 cells taken on day 3 of the **CTH-BzPEG** hydrogel drug release assay with doxorubicin (0.5 mg/mL): A) untreated cells, B) cells treated with the hydrogel without doxorubicin and C) cells treated with the hydrogel containing doxorubicin. Scale bar: 30 μm.
